# The relationship between physical activity, physical fitness and overweight in adolescents: a systematic review of studies published in or after 2000

**DOI:** 10.1186/1471-2431-13-19

**Published:** 2013-02-01

**Authors:** Annette Rauner, Filip Mess, Alexander Woll

**Affiliations:** 1 Division of Sport Science, University of Konstanz, Konstanz, Germany; 2 Department of Sport and Sport Science, Karlsruhe Institute of Technology, Karlsruhe, Germany

**Keywords:** Physical activity, Cardiorespiratory fitness, Motor fitness, Overweight, Obesity, Adolescent, Youth, Cross-sectional studies, Longitudinal studies

## Abstract

**Background:**

Not only in adults but also in children and adolescents, obesity increases the risk for several health disorders. In turn, many factors including genetic variations and environmental influences (e.g. physical activity) increase the risk of obesity. For instance, 25 to 40 percent of people inherit a predisposition for a high body mass index (BMI). The purpose of this systematic review was to summarize current cross-sectional and longitudinal studies on physical activity, fitness and overweight in adolescents and to identify mediator and moderator effects by evaluating the interaction between these three parameters.

**Methods:**

The electronic academic databases PubMed, SportDiscus, WEB OF KNOWLEDGE and Ovid were searched for studies on physical activity, fitness and overweight in adolescents aged 11 to 19 years (cross-sectional studies) and in adolescents up to 23 years old (longitudinal studies) published in English in or after 2000.

**Results:**

Twelve cross-sectional and two longitudinal studies were included. Only four studies analyzed the interaction among physical activity, fitness and overweight in adolescents and reported inconsistent results. All other studies analyzed the relationship between either physical activity and overweight, or between fitness and overweight. Overweight—here including obesity—was inversely related to physical activity. Similarly, all studies reported inverse relations between physical fitness and overweight. Mediator and moderator effects were detected in the interrelationship of BMI, fitness and physical activity. Overall, a distinction of excessive body weight as cause or effect of low levels of physical activity and fitness is lacking.

**Conclusions:**

The small number of studies on the interrelationship of BMI, fitness and physical activity emphasizes the need for longitudinal studies that would reveal 1) the causality between physical activity and overweight / fitness and overweight and 2) the causal interrelationships among overweight, physical activity and fitness. These results must be carefully interpreted given the lack of distinction between self-reported and objective physical activity and that studies analyzing the metabolic syndrome or cardiovascular disease were not considered. The importance of physical activity or fitness in predicting overweight remains unknown.

## Background

Overweight and obesity has been called a global epidemic by the World Health Organization [1]. The prevalence of overweight and obesity is especially dramatic in economically developed countries [2] and not only in adults but also in children and adolescents. In Germany for instance, 17% of adolescents aged 14 to 17 years are overweight and nearly 9% are obese [3]. Similarly, in the United States, 18% of adolescents aged 12 to 19 years were obese in 2007/2008 [4]. In accordance with the literature [5-9], the term overweight includes obesity in this review.

Several health conditions and disorders have been attributed to being overweight in children and adolescents [10]. For instance, overweight children and adolescents are more likely to suffer from cardiovascular, metabolic, pulmonary, skeletal or psychosocial disorders [11]. Even if these conditions or disorders are not manifested during childhood, being overweight in childhood increases the risk of illness in adulthood [10]. Hence, it is critical to identify risk factors for overweight in children and adolescents and to address overweight during childhood and adolescence.

Being overweight may originate from many different factors ranging from environmental influences to genetic variations [12]. The heritability of predisposition for a high body mass index (BMI) or body fat content is between 25 and 40% [13], which suggests that other factors such as environmental factors may also play a critical role. According to Bouchard et al. [13], both the family environment and genetic predisposition influence the development of body fat content and distribution. Other important factors include lifestyle factors such as physical activity (PA), nonsmoking, high-quality diet, sedentary activities and normal weight [14]. Lifestyle factors are also important in the description of the obesogenic environment that is based on the four pillars family, sport and leisure time, eating behavior and social education [15].

Several epidemiological and intervention studies [16,17] have identified the role of physical activity and physical fitness for overweight in children and adolescents, and hence we focused on the role of sport during leisure time. Previous reviews [18-20] provided an overview of studies on the relationship either between physical activity and overweight or between fitness and overweight in children or adolescents. Despite of the influence of physical activity and fitness similarly on health outcomes including overweight, to date results of studies on the interaction between all three parameters have not been synthesized although these parameters cannot be considered independently [21]. In addition, most reviews omitted studies on adolescents and young adults or did not include longitudinal studies.

The purpose of this systematic review was to provide an overview of cross-sectional and longitudinal studies published in or after 2000 on physical activity, fitness, and overweight in adolescents, and to identify mediator and moderator effects in the interrelationship among these three parameters particularly considering gender differences because of the significant differences in these parameters between boys and girls [22].

### Definitions

*Physical activity* comprises all modes of movement caused by muscle activity resulting in increased energy expenditure [19,23].

*Physical fitness* consists of the three components muscle strength, endurance and motor ability, and is a prerequisite for completing daily activities without fatigue and for participating in leisure time activities [24].

*Overweight and obesity* are defined as abnormally high fat content that may impair health and as high bodyweight (exceeding the standard measure) caused by an increased fat consumption [11].

## Methods

### Data collection

One author (AR) searched the electronic academic databases *PubMed*, *SportDiscus*, *web of knowledge* and *Ovid* for relevant studies. The following search terms were used: [“physical activity” or “fitness” or “exercise”] and [“obes*” or “overweight” or “weight gain” or “BMI”] and [“youth” or “adolescents”]. The data collection was completed in October 2011 (date of last search: 28/10/2011).

**Figure 1:**
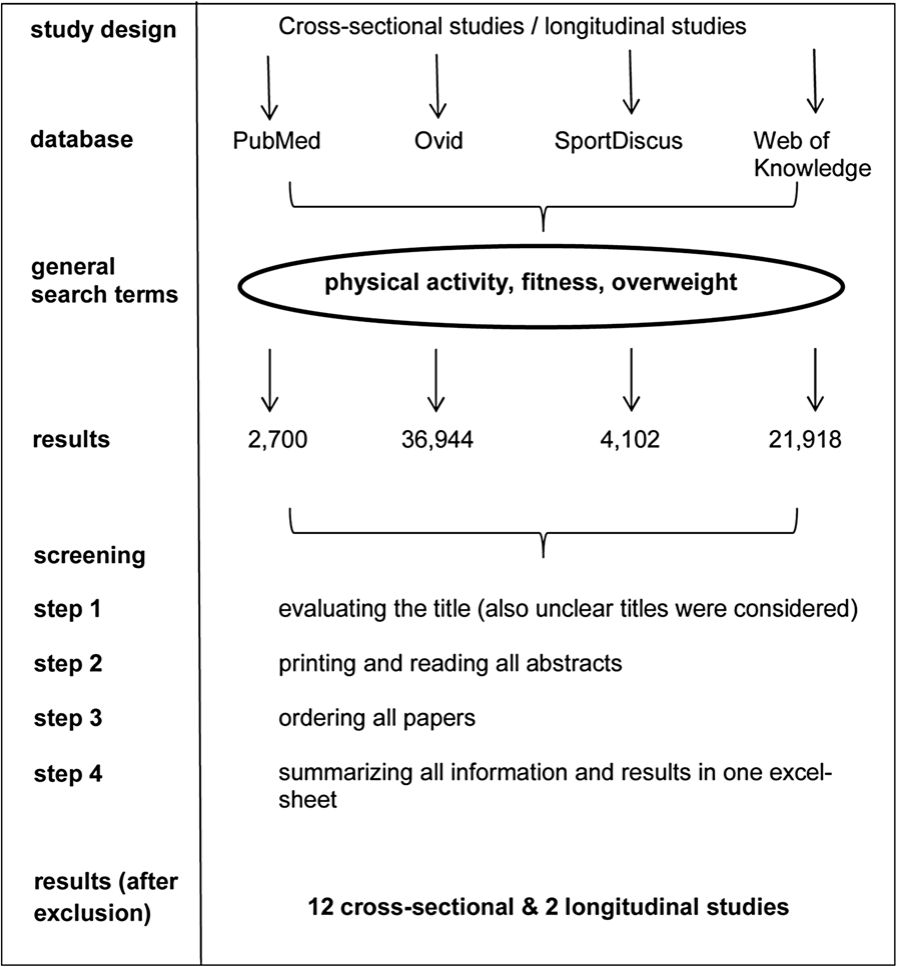
Diagram illustrating the four-step search strategy.

The four-step search strategy is illustrated in Figure 1. In step 1, articles were screened based on title; in step 2, articles were selected based on the abstracts; in step 3 full versions of included articles were ordered; and all information was summarized in step 4. The abstracts formed an important element of the selection process and were used as decisive criterion for ordering full versions of the articles.

### Inclusion criteria

We included only cross-sectional studies with study populations (prospective cohort studies with random sample) aged 11 to 19 years and longitudinal studies with an upper age limit of 23 years. However, two cross-sectional studies with a target group aged 7 to 12 years were also included because the age range of the study population overlapped with the target age range and the results were comparable with findings of other included studies. The search was limited to articles published in or after 2000 with physical activity and physical fitness as exercise components because research on childhood and adolescence overweight and its interaction with physical activity and physical fitness has greatly increased since 2000. Only articles published in English were included.

### Exclusion criteria

Intervention studies, clinical trials, overviews and summarizing reviews and studies that did not analyze all three parameters physical activity, physical fitness (motor or cardiorespiratory fitness) and overweight were excluded.

## Results

The literature search of the four databases yielded 65,664 hits (Figure 1). Twelve cross-sectional and two longitudinal studies fulfilled all criteria and were included after the screening process.

### Measurements

#### Assessment of overweight

All included cross-sectional studies used BMI as measurement of overweight or obesity [6-9,25-32]. Height and weight were quantified in ten studies [7,8,25-32] and self-reported in two studies [6,9]. In both longitudinal studies, BMI was used to determine overweight or obesity [5,33]. In two studies, waist circumference was also determined [26,30], and in five studies [4,29-32] skinfold thickness was measured. Only one study used bioelectrical impedance analysis (BIA) [8] and one study used Dual Energy X-ray Absorptiometry (DXA) [32] for determining overweight or obesity.

#### Measurement of physical fitness

Four cross-sectional studies included both cardiorespiratory and motor fitness [6,28,29,31]. The other eight cross-sectional studies [7-9,25-27,30,32] and the two longitudinal studies [5,33] assessed only cardiorespiratory fitness.

#### Measurement of physical activity

The included studies measured physical activity using several different methods. Five studies used objective measurements such as accelerometry [7-9,32] and pedometry [30]. Ten studies (eight cross-sectional and two longitudinal studies) used subjective measurements derived from questionnaires with items relating to the setting (at school, outside school; divided into leisure time physical activities at sport clubs and leisure time physical activities outside of sports clubs) and intensity of physical activities [6,8,25-29,31]. Only one study collected both objective and subjective data on physical activity [8]. Most studies analyzed the relationship between overweight, physical activity and physical fitness using analyses of variance (ANOVA) and (linear and logistic) regression analysis.

### Associations between physical activity, physical fitness and overweight in cross-sectional studies

Twelve studies met the inclusion criteria of this review. While all twelve studies assessed physical activity, physical fitness and overweight, only four studies analyzed the interaction among these three parameters. Because some studies did not report actual data but only an interpretation of their findings, statistical parameters could only be included for some studies. The results of all included studies are summarized in (see Additional file 1: Table S1). For completeness, we also report the results of those studies that assessed all three parameters but not their interaction. Throughout this article, we distinguish between genders because of the significant differences in physical activity, physical fitness and overweight between boys and girls [22]. The effects of physical activity and physical fitness on overweight and the strength of the association between physical activity, physical fitness and overweight by gender are summarized in (see Additional file 2: Table S2). We defined four categories based on the results of the statistical tests (p>0.05; p<0.05; p<0.01; p<0.001) and interpreted the associations regarding overweight: for instance, a positive relationship between fat free mass and physical activity was interpreted as a negative association between overweight and physical activity and the corresponding statistical result transcribed by the corresponding statistical result. The results in this table clearly show a stronger relationship and a more pronounced gender effect on the relationship between physical fitness and overweight than between physical activity and overweight (see Additional file 2: Table S2).

#### Relationship between physical activity, physical fitness and overweight

Because the statistical data evaluation in the included studies was heterogeneous, the central outcomes of all studies cannot be summarized, and hence we present the results of each study. Ortega et al. [26] reported a higher BMI in adolescents with lower cardiorespiratory fitness independent of their sedentary and leisure time activities (physical activities outside school). In boys and girls, BMI was negatively correlated with cardiorespiratory fitness independent of their leisure-time physical activity and sedentary activities (boys: p=0.006; girls: p<0.001). Similarly, cardiorespiratory fitness was inversely related to waist circumference (boys: p=0.001; girls: p=0.005) independent of physical activity. Up to 10% of variance in waist circumference in boys and 18% of variance in waist circumference in girls was explained by their sedentary activities (television viewing and video/computer time). Variability in cardiorespiratory fitness explained up to 13% of the variance in waist circumference in boys and up to 16% in girls.

In contrast, Fogelholm et al. [6] found that variance in physical activity better explained the variability in physical fitness (ß-coefficients between −0.33 and 0.49) than that in overweight (ß-coefficients between −0.27 and 0.24). The associations between overweight and physical activity and between physical activity and physical fitness were comparable for both genders. The intensity of physical activity and being overweight predicted physical fitness in adolescents. Fogelholm et al. [6] described that physically active persons who are overweight cannot achieve better physical fitness values because of the negative association between being overweight and physical fitness. Thus, overweight acts as a mediator for the relationship between physical activity and physical fitness.

In another study, Ortega [9] showed that cardiorespiratory fitness influences the association between being overweight and physical activity. Hence, cardiorespiratory fitness acts as a moderator for the relationship between overweight and physical activity. The association between physical activity, physical fitness and overweight did not differ between genders. Lohman et al. [32] reported that girls with high levels of physical activity (one standard deviation above the mean) and average body composition (fat free and fat mass) had a higher physical fitness level (+3.5%) and girls with low levels of physical activity (one standard deviation below the mean) and average body composition have a lower physical fitness level (−3.5%) compared with that of girls with average levels of physical activity and average fat free and fat mass.

#### Relationship between physical fitness and overweight

(see Additional file 2: Table S2) shows that all studies showed an inverse relationship between physical fitness and overweight and overweight and physical fitness respectively, except for Huotari et al. [27], where no data were available. In two studies, cardiorespiratory fitness [6,31] was more strongly related to overweight than motor fitness, and two studies [28,29] showed a stronger relationship between BMI and cardiorespiratory fitness than between BMI and motor fitness. Interpreting and comparing these results is difficult because these studies used different analytic strategies and measurement instruments. The four studies [6,7,28,31] used three to seven different tests for measuring several aspects of motor capacity.

All twelve studies reported an inverse relationship between cardiorespiratory fitness and overweight. Seven studies [6,7,26,28-31] used shuttle run tests, and one study each used the cooper test [8], a submaximal treadmill test [25], the PWC 170 [32], the maximal cycle test [9] or the 2000-m (boys) and 1500-m (girls) running test for assessing cardiorespiratory fitness.

Pate et al. [25] found no difference in the relationship between physical fitness and BMI between genders, and Ortega et al. [26] observed a similar relationship between overweight and physical fitness for both genders. In addition, BMI adjusted by waist circumference was significantly negatively associated with cardiorespiratory fitness only in overweight boys (p≤0.05) but not in normal weight adolescents and overweight girls. Cardiorespiratory fitness was inversely associated with BMI in boys and in girls (p<0.001) and with waist circumference (boys: p=0.001; girls: p=0.005). Variance in cardiorespiratory fitness explained up to 13% of variability in waist circumference in boys and up to 16% in girls. In addition, the comparison of cohorts collected in 1976 and 2001 by Huotari et al. [27] confirmed these findings. In girls, the influence of BMI on cardiorespiratory fitness was smaller (ß=−0.42, p<0.001, R^2^=0.165) than that in boys (ß=−0.36, p<0.001, R^2^=0.127) in the 2001 study. In comparison, in the 1976 study no significant relationship between BMI and cardiorespiratory fitness was found for girls or boys. The results by Gonzales-Suarez and colleagues [28] were not stratified by gender. Fogelholm et al. [6] did not find significant differences in ß-coefficients for endurance capacity between genders.

Ara et al. [29] reported that skinfold thickness was most strongly related to cardiorespiratory fitness in both boys and girls, and the ß-coefficient for this relationship was greater in boys (ß=−3.334; p<0.001) than in girls (ß=−2.571; p<0.001). The next strongest predictors of cardiorespiratory fitness were truncal subcutaneous fat (boys: ß=−1.78, p<0.001; girls: ß=−1.77, p<0.001) and BMI (boys: ß=−0.047, p<0.001; girls: ß=−0.059, p<0.001).

The results by Deforche et al. [31] are comparable to those reported by Aires et al. [7], and the endurance capacity in obese boys was higher than that in obese girls (F=22.5; p<0.001). Haerens et al. [8] analyzed the difference in overweight and cardiorespiratory fitness by gender and detected a significant (F=6.08; p≤0.05) difference in running capacity between overweight boys and girls. Ortega [9] reported an inverse association between waist circumference and cardiorespiratory fitness without a significant gender effect. Fogelholm [6] showed marginally stronger relationships between ball skills (ß_boys_=−.12, p<0.001; ß_girls_=−0.10, p=0.003), jumping back and forth (ß_boys_=−0.17, p<0.001; ß_girls_=−0.14, p<0.001) and five-jump (ß_boys_=−0.27, p<0.001; ß_girls_=−0.26, p<0.001) and overweight in boys than in girls. In comparison, the influence of overweight on number of sit-ups (ß_boys_=−0.20, p<0.001; ß_girls_=−0.21, p<0.001) and the coordination test (ß_boys_=−0.22, p<0.001; ß_girls_=−0.24, p<0.001) was stronger in girls than in boys. Deforche et al. [31] found a significant interaction between gender and obesity in the sit and reach test (F=4.3; p<0.05), bent-arm-hang (F=45.8; p<0.001) and endurance shuttle run (F=22.5; p<0.001). Ng et al. [30] did not stratify weight groups by gender, Lohman et al. [32] only included females in their study, and Gonzales-Suarez et al. [28] did not perform a separate analysis of the relationship between overweight and motor fitness for genders.

#### Relationship between physical activity and overweight

In comparison to physical fitness, the relationship between physical activity and overweight is less clear (see Additional file 2). Three studies collected physical activity data but did not analyze the relation between physical activity and overweight [6,25,27]. Six studies did not find any relationships between overweight and physical activity [7,26,29-31,34]. Two studies [28,32] analyzed relations between physical activity and overweight and between overweight and physical activity, respectively. Lohman et al. [32] found a negative significant correlation between BMI and physical activity, whereas Gonzales et al. [28] did not find differences between BMI and physical activity scores in overweight and normal youth.

Objective and subjective measurement instruments yielded comparable results. While one study detected a relationship between objectively measured physical activity and overweight [32], two studies did not find any relationship between overweight and objectively measured physical activity [7,30]. We found similar results for studies using subjective measurement instruments. One study reported a significant relationship between overweight and subjectively measured physical activity [28], and three studies did not find relationships between overweight and subjectively measured physical activity [26,29] and subjectively measured physical activity and overweight [31]. Two studies [8,9] used both objective and subjective instruments for assessing physical activity. While Ortega et al. [9] did not find any relationship between overweight and physical activity, Haerens [8] detected significant relationships between overweight and physical activity dependent of the method of data evaluation. Similarly, categorized physical activity (active versus non-active) was not related to overweight [29]. In comparison, the intensity of physical activity was related to overweight [7,8].

Five studies [8,26,28,29,31] analyzed differences in the relationship between physical activity and overweight between genders. Two studies [26,29] found a stronger relationship between physical activity and overweight for boys than for girls. In contrast, three studies [8,28,31] did not find a gender effect on the relationship between (total) physical activity and overweight. Ortega and colleagues [26] performed separate median value comparisons between BMI and waist circumference, and activity pattern and cardiorespiratory fitness by gender. The strongest relationship in boys (p=0.006) was that between waist circumference and sedentary activities was. In girls, the strongest association was that between waist circumference and active commuting to school (no information was provided on type of active commuting; p=0.002). A significant relationship between BMI and sedentary activities (≤ 2 hours; ß=−0.72; p=0.043) was found only in boys, whereas waist circumference was negatively associated with sedentary activities (≤ 2 hours) in boys (ß=−2.46; p=0.024) and in girls (ß=−1.47; p=0.028). Up to 10% of variance in waist circumference in boys and up to 18% in girls were explained by variability in sedentary activities. In contrast, Gonzales-Suarez [28] did not find an effect of gender on the relationship between being overweight and physical activity. Ara et al. [29] analyzed the differences in weight (measured using various methods) between active and non-active adolescents. BMI was higher in active boys than in non-active boys (p=0.05), and the sum of skin fold test scores was slightly higher in active than in non-active boys. In contrast, while fat mass was lower in active girls than in non-active girls (p<0.05), both groups had comparable BMI. Deforche et al. [31] reported a higher sport index in non-obese boys compared to obese boys (F=3.7; p<0.05), and a comparable sport index in obese and non-obese girls. Aires et al. [7] did not report a gender specific analysis. Haerens et al. [8] did not find significant differences between body weight groups in objectively (F=0.08; p>0.05) or subjectively (F=0.03; p>0.05) measured total physical activity analyzed by gender. However, moderate physical activity significantly differed between boys and girls (F=4.25; p≤0.001). The results for overfat (measured via skinfold thickness) and normal fat boys and girls were comparable. Objectively (F= 0.47; p>0.05) and subjectively (F=2.13; p>0.05) measured total physical activity, light physical activity (F= 0.18; p>0.05) and moderate physical activity (F=1.4; p>0.05) did not differ significantly between overfat and normal fat boys and girls.

### Associations between physical activity, fitness and overweight in longitudinal studies

Two longitudinal studies captured physical activity, physical fitness and overweight.

#### Relationship between physical activity, physical fitness and overweight

Both longitudinal studies [5,33] analyzed only the relationship between physical activity and overweight and between physical fitness and overweight and not the interaction among all three parameters. However, separate analyses by Aires et al. [33] showed that while physical activity influenced cardiorespiratory fitness and cardiorespiratory fitness influenced BMI, BMI was not related to physical activity. Therefore cardiorespiratory fitness acts as a mediator in the relationship between physical activity and BMI.

#### Relationship between physical fitness and overweight

He et al. [5] and Aires et al. [33] reported an inverse relationship between BMI and physical fitness and between physical fitness and BMI respectively. Subjects with a low fitness level at baseline had a higher risk of becoming overweight or obese compared to those who had high initial fitness levels (data not shown) [5].

Aires et al. [33] did not report a potential gender difference. In contrast, He [5] found that boys with low fitness at baseline were more likely to be overweight 3-years later than girls (boys: OR=8.71, p<0.001; girls: OR=6.87, p=0.055).

#### Relationship between physical activity and overweight

The questionnaire used by Aires et al. [33] provided information on sedentary activities. Adolescents with low physical activity levels did not experience a significant increase in BMI over time [33]. Similarly, He et al. [5] did not reveal significant associations between changes in BMI and physical activity. None of the studies investigated the influence of gender on the relationship between physical activity and overweight.

## Discussion

The purpose of this systematic review was to provide an overview of cross-sectional and longitudinal studies published in or after 2000 on physical activity, physical fitness and overweight in adolescents, and to identify mediator and moderator effects in the interrelationship among these three parameters particularly considering gender differences. Objectivity of self-reported physical activity has been questioned because of potential over- or underestimation [32] and thus should be considered with caution. However, because only few studies examined the interaction between physical activity, physical fitness and overweight, we combined results of objectively and subjectively assessed physical activity.

To the best of our knowledge, this article is the first review on the interrelationship between physical activity, physical fitness and overweight, and hence our results cannot be related to the literature or to other study populations. Synthesizing the interaction between all three parameters was difficult because only four studies specifically investigated this interaction. While the literature reported inconsistent results, all studies showed an interaction between these parameters. Several studies [6,9,26,32] confirmed that physical activity and physical fitness are equally important for health [21]. In the following the results will be discussed with reviews analyzing only the relationship between two parameters, because no comparable reviews (reviews analyzing the interaction) were found.

The different strengths of the correlations between the three parameters may be at least in part attributed to the different measurements of physical activity. For instance, two studies [6,26] assessed physical activity via questionnaire, one via accelerometer [32] and one via activity monitor and questionnaire [9], and the collection period of objectively measured physical activity ranged from three [9] to six [32] days. In addition, the two studies that measured physical activity subjectively omitted reporting details on their measurement instruments. Further, Ortega et al. [26] measured physical activity outside of school for only four days. While Fogelholm [6] measured the activity during leisure-time in and outside of sports clubs, they only reported frequency and duration and not intensity or setting of physical activity. Hence, reliable and valid questionnaires assessing frequency, duration, intensity and setting of the different physical activities are still needed [35] especially because, for instance, intensity is an important aspect in overweight prevention [36]. Interestingly, studies that used unspecific measurement instruments for physical activity reported weak or no relationships between physical activity and overweight [9,26,29]. The poor quality of physical activity measurement instruments may also explain the stronger influence of cardiorespiratory fitness than that of physical activity on overweight. The main limitation of subjective measurement instruments is potential over- and underestimation of physical activity [32]. In comparison, objective measurement instruments for physical activity can only capture specific activities and require a high effort by the participants. For instance, subjects have to regularly wear the accelerometers or pedometers for extended periods of time and on different days.

The data on the relationship between physical activity and overweight are inconsistent. Specifically, the different levels of physical activity (measured by objective measurement methods) showed different relationships to overweight. In addition, the effect of gender on the relationship between physical activity and overweight was inconsistent. While Deforche et al. [31], Haerens et al. [8] and Gonzales-Suarez et al. [28] reported no gender effect on the relationship between overweight and physical activity, other studies [26,29] revealed that gender affected the relationship between overweight and physical activity but that this association depended on the anthropometric measurement method used to measure overweight. Similar to our observations in adolescents, Must et al. [19] found inconsistent results in children with a higher tendency to an inverse relationship between physical activity level and overweight in *cross-sectional studies* and differences in the relationship between physical activity and overweight between boys and girls emphasizing the inconsistent state of research not only in adolescents but also in children. The previously discussed large number and poor quality of methods for measuring physical activity might explain this observation. In addition, these results show that capturing physical activity in youth is difficult. In a review of cross-sectional studies that analyzed self-reported and objectively measured physical activity in overweight children and adolescents, Winkler et al. [36] reported inconsistent results and that the intensity of physical activity played a critical role independent of age and gender. In addition, Winkler et al. [36] reported that physical activity was related to overweight in two *longitudinal studies*, which contradicts the results of the two longitudinal studies [5,33] included in our review that found no relationships between physical activity and overweight. In contrast, Must et al. [19] reviewed longitudinal studies and reported comparable results to our findings in longitudinal studies. Similarly to adolescents, the results in children are inconsistent and low physical activity level was not related to changes in BMI [5,19,33]. However, according to Must et al. [19], most cross-sectional and longitudinal studies showed no relationships between physical inactivity and overweight in adolescents and inconsistent gender specific results.

All studies included in our review observed inverse relationships between physical fitness and overweight. Because of the different measurement instruments for cardiorespiratory fitness used in these studies (shuttle run: [5-7,26,28-31,33], maximal treadmill test: [25], maximal cycle test: [9], cooper test: [8], PWC 170: [32], 2,000/1,500 m: [27]), final comparisons are difficult. Adolescents with lower cardiorespiratory fitness were more likely to be overweight or obese than those with high cardiorespiratory fitness [5-9,25,26,28-32]. However, gender influenced the relationship between overweight and cardiorespiratory fitness. These results are in agreement with the results of other studies including those reported by Ostojic et al. [37]. Similar results were observed for motor fitness and overweight. The measurement instruments were also inconsistent in motor fitness (Eurofit [29,31]: two studies; unknown [6,28]: two studies). While motor fitness in overweight and obese adolescents was lower than that in normal weight adolescents [6,28,29,31,33], the influence of gender on the relationship between motor fitness and overweight was heterogeneous.

Interestingly, some studies [6,25,27,31-33] included weight as independent parameter in their statistical models while other studies [5,7-9,26,28-30] used weight as dependent parameter. This observation illustrates that the causality between physical activity and overweight and between physical fitness and overweight is still unclear. For instance, Metcalf et al. [38] suggested that overweight influences level of physical activity but not vice versa. Similar data for the causal relationship between physical fitness and overweight are not available. Hence, future longitudinal studies are warranted to tease out this causal relationship. Furthermore, additional longitudinal analyses are necessary to determine the interrelationship (mediator or moderator effect) between physical activity, physical fitness and overweight which has important implications for public health policy making and developing optimal obesity prevention or treatment programs.

### Limitations

Because of the small number of studies the results were not categorized based on objective or subjective physical activity measurement. In addition, studies on metabolic syndrome or cardiovascular diseases were not included (even if physical activity, physical fitness and overweight measures were used), and only studies with the primary goal of analyzing the relationship between the three parameters were included.

## Conclusion

The small number of longitudinal studies emphasizes the lack of longitudinal research, and further prospective studies are necessary for determining cause and effect and the type (correlation, mediator and moderator effect) of the interrelationship among physical activity, physical fitness and overweight.

Overall, a concluding evaluation is difficult because several studies did not state effect or effect size and hence the reported information on significant relationships should be interpreted with caution. In addition, the studies used different methods to measure physical activity, and the objectivity of self-reported physical activity is questionable [39] and may result in over- or underestimation [32].

## Abbreviations

BMI: Body mass index; PWC: Physical work capacity.

## Competing interests

The authors declare no competing interests.

## Authors’ contribution

AR performed the literature search and drafted the manuscript. FM revised the manuscript and supported the process of writing the manuscript. AW gave the final approval of the version to be published. All authors read and approved the final manuscript.

## Pre-publication history

The pre-publication history for this paper can be accessed here:

http://www.biomedcentral.com/1471-2431/13/19/prepub

## Supplementary Material

Additional file 1 **Table S1.** Cross-sectional and longitudinal studies examining the relation between physical activity, physical fitness and overweight in adolescents. The Table 1 illustrates all included studies (study population, study design, measurements, main results). Click here for file

Additional file 2 **Table S2.** Overview of the strength of the different associations. Table 2 gives an overview of the different effects from physical activity and physical fitness on overweight and the interaction between physical activity, physical fitness and overweight respectively from each study. Click here for file
