# Supplementary material for: The relationship between physical activity, physical fitness and overweight in adolescents: a systematic review of studies published in or after 2000
Source: BMC Pediatr. 2013 Feb 1;13:19. doi: 10.1186/1471-2431-13-19 (PMC3571910; doi:10.1186/1471-2431-13-19)
Supplement: Additional file 1 — Table S1. Cross-sectional and longitudinal studies examining the relation between physical activity, physical fitness and overweight in adolescents. The Table 1 illustrates all included studies (study population, study design, measurements, main results). [file 1471-2431-13-19-S1.pdf]

Table 1: Cross-sectional and longitudinal studies examining the relation between physical activity, physical fitness and overweight in adolescents.

Reference	Study design	Anthropo-metrics	Cardio-respiratory fitness	Motor fitness	Objective physical activity	Subjective physical activity	Results
Deforche et al., 2003 [31]	cross-sectional; age: 12–18 years, N=3,214	BMI [41], 5 skinfold thickness	shuttle run	10x5m shuttle run, standing broad jump, sit and reach, flamingo balance, plate tapping, sit ups, bent arm hang	-	sport index and leisure-time index	Fitness: speed shuttle run: sign. association with obesity (F= 134.4; p<0.001).<br>endurance shuttle run: sign. association with obesity (F= 359.3; p<0.001).<br>handgrip: sign. association with obesity (F= 40.9; p<0.001).<br>plate tapping and sit and reach: similar for both groups.<br>PA: no differences in leisure-time index.
Ng et al., 2006 [30]	cross-sectional; age: 9–12 years, N=82	BMI [41], WC, 5 skinfold thickness	shuttle run	-	pedometers (2 days)	-	Fitness: sign. lower fitness scores in obese children (F= 21.0; p<0.001).<br>PA: not sign. among body mass groups.
Pate et al., 2006 [25]	cross-sectional; age: 12 to 19 years; N= 3,287	BMI [42]	submaximal treadmill exercise test	-	-	MET sedentary activities	Fitness: sign. higher CRF levels in normal weight than in overweight youth (p<0.001).<br>PA: no analyses.

Sign. = significant; PA = physical activity; CRF = cardiorespiratory fitness; BMI = body mass index; MPA = moderate physical activity; MVPA = moderate-to-vigorous physical activity; VPA = vigorous physical activity; VVPA = very vigorous physical activity, WC = waist circumference; WHR = waist to hip ratio; LTPA = leisure-time physical activity; SSF = skinfold thickness; STF = truncal subcutaneous fat; PWC = physical work capacity

Reference	Study design	Anthropo-metrics	Cardio-respiratory fitness	Motor fitness	Objective physical activity	Subjective physical activity	Results
Ara et al., 2007 [29]	cross-sectional; age: 7–12 years; N=1,068	BMI [41], 6 skinfold thickness	shuttle run	10x5m shuttle run, flexibility, standing long jump, sit ups, handgrip, bent arm hang	-	categorized into active/not active	Fitness: low correlations between fat mass and fitness parameters (r< 0.32), (except for CRF: r between 0.48 and 0.51; p<0.01 and bent arm hang: r between 0.36 and 0.40; p<0.01). CRF: strongest correlation with BMI and fat mass compared to PA)<br>PA: trend to lower values in skinfold thicknesses in the active group (p= 0.07).
Haerens et al. 2007 [8]	cross-sectional; age: 11–13 years; N=222	BMI [41], BIA	Cooper test	-	accelero-meter	active transportation index, sports index, physical activity at school index, leisure-time physical activity index	Fitness: main effect of weight status (F= 36.63, p<0.001) in the CRF. Sign. differences in the running capacity test (F= 46.17; p<0.001).<br>PA: sign. differences in leisure time (F= 4.48; p<0.05); no sign. differences in total PA levels, school PA, active transportation and sport. Sign. association between overweight and MPA (F= 6.13; p= ≤ 0.05). Sign. association between overweight and MVPA (F= 6.55; p≤0.05). No sign. associations between overweight and total PA, active transportation, sport, school PA und LTPA.

Sign. = significant; PA = physical activity; CRF = cardiorespiratory fitness; BMI = body mass index; MPA = moderate physical activity; MVPA = moderate-to-vigorous physical activity; VPA = vigorous physical activity; VVPA = very vigorous physical activity, WC = waist circumference; WHR = waist to hip ratio; LTPA = leisure-time physical activity; SSF = skinfold thickness; STF = truncal subcutaneous fat; PWC = physical work capacity

Reference	Study design	Anthropo-metrics	Cardio-respiratory fitness	Motor fitness	Objective physical activity	Subjective physical activity	Results
Ortega et al., 2007 [26]	cross-sectional; age: 13–18.5 years; N=2,859	BMI [41]; WC	shuttle run	-	-	leisure-time PA index, active commuting to school, MET sedentary activities	Fitness: inverse correlation between BMI-adjusted WC and CRF ($p \leq 0.05$). PA: no sign. associations between BMI($p>0.05$) or WC ($p>0.05$) and LTPA. Fitness & PA: inverse correlation between WC / BMI and CRF, independent of sedentary activities or PA (data not shown). Association between lower obesity risk (when measured by WC) and fitness / sedentary activities, not in PA.
Fogelholm et al., 2008 [6]	cross-sectional; age: 15–16 years; N=2,266	BMI [41]	shuttle run	sit ups, sit and reach, back and forth jumping, five jump, ball skill test, coordination test	-	frequency of PA	Fitness: significant effect of overweight for all tests, excluding sit and reach ($p<0.002$). Sign. association between five-jump, endurance shuttle run and fitness index and overweight (ß-coefficients: -0.02 to -0.26; $p<0.001$; R^2: 0.14 to 0.32). PA: no differences between PA level and weight groups Fitness +PA: poorer results in all fitness tests for overweight regardless of PA levels. PA explained more variance in fitness test results than BMI.

Sign. = significant; PA = physical activity; CRF = cardiorespiratory fitness; BMI = body mass index; MPA = moderate physical activity; MVPA = moderate-to-vigorous physical activity; VPA = vigorous physical activity; VVPA = very vigorous physical activity, WC = waist circumference; WHR = waist to hip ratio; LTPA = leisure-time physical activity; SSF = skinfold thickness; STF = truncal subcutaneous fat; PWC = physical work capacity

Reference	Study design	Anthropo-metrics	Cardio-respiratory fitness	Motor fitness	Objective physical activity	Subjective physical activity	Results
Lohman et al., 2008 [32]	cross-sectional; age: 14 years; N=1,440	high and weight, 1 skinfold thickness, percent fat (DXA, BMI, skinfold thicknesses)	PWC 170	-	actiGraph	-	Fitness: correlation with PWC 170: fat free mass (r= 0.26 p<0.05) fat mass (r= 0.16, p<0.5), body composition (r= -0.36 to -0.54). PA: Inverse correlation between MVPA and BMI and MVPA and fat mass (r= -0.14 and r=-0.12, p<0.05). Fitness + PA: high level of PA, average %body fat: high fitness level. Low level of PA, average %body fat: low fitness level.
Aires et al., 2010 [7]	cross-sectional; age: 11–18 years; N=111	BMI [41]	shuttle run	-	accelero-meter	-	Fitness: overweight youth: sign. lower CRF levels (p<0.05); BMI inverse correlation with CRF (r=- 0.2, p<0.05); CRF pos. correlated with VPA (r=0.39, p<0.001), VVPA (r= 0.28, p<0.001), the amount of PA (r= 0.28, p<0.001). Higher levels of CRF: lower relative risk of being overweight/obese (OR=0.968 (0.939 to 0.998), p=0.037). PA: no associations between BMI level and total amount of PA or PA intensity.
Huotari et al., 2010 [27]	cross-sectional; age: 13–18 years; N=558 (2001); N=717 (1976)	BMI [41]	2,000-m running test (boys), 1,500-m running test (girls)	-	-	frequency per week of PA outside school	No separate analyses for PA and CRF in relation to association with overweight, but regression model shows that LTPA (ß: 0.23 to 0.30; p<0.001) and BMI (ß: -0.10 to -0.42; p<0.001) significant predictors of CRF.

Sign. = significant; PA = physical activity; CRF = cardiorespiratory fitness; BMI = body mass index; MPA = moderate physical activity; MVPA = moderate-to-vigorous physical activity; VPA = vigorous physical activity; VVPA = very vigorous physical activity, WC = waist circumference; WHR = waist to hip ratio; LTPA = leisure-time physical activity; SSF = skinfold thickness; STF = truncal subcutaneous fat; PWC = physical work capacity

Reference	Study design	Anthropo-metrics	Cardio-respiratory fitness	Motor fitness	Objective physical activity	Subjective physical activity	Results
Ortega et al., 2010 [9]	cross-sectional; age: 15 years; N=518	BMI [43], WC, skinfold thickness	maximal cycle test	-	activity monitor	sedentary activities (TV time)	Fitness: negative association between WC and CRF (p=0.002). PA: no associations between PA parameters and WC. Fitness+ PA: no association between WC and VPA / total PA, but significant interactions with CRF (CRF and VPA: ß=0.01, p=0.005; CRF and total PA: ß=0.01; p=0.02); inverse association between WC and low CRF level and VPA (ß=-0.10; p=0.04). No sign. associations between total PA and WC (ß=-0.08; p=0.08); high fitness level: all PA parameters positively associated with WC (ß: 0.11 to 0.25; p=0.025 to p<0.001); prevalence of being overweight, having an excess of total fatness and having a high risk WC were sign. lower in the high CRF group than in the low CRF group (all p≤0.05). CRF: component of the model: moderate PA and MVPA sign. positively associated with WC (p=0.01 and p=0.03).

Sign. = significant; PA = physical activity; CRF = cardiorespiratory fitness; BMI = body mass index; MPA = moderate physical activity; MVPA = moderate-to-vigorous physical activity; VPA = vigorous physical activity; VVPA = very vigorous physical activity, WC = waist circumference; WHR = waist to hip ratio; LTPA = leisure-time physical activity; SSF = skinfold thickness; STF = truncal subcutaneous fat; PWC = physical work capacity

Reference	Study design	Anthropo-metrics	Cardio-respiratory fitness	Motor fitness	Objective physical activity	Subjective physical activity	Results
Gonzales-Suarez et al., 2011 [28]	cross-sectional; age: 11–12 years; N=4,600	BMI [41]	shuttle run	standing broad jump, 50-m sprint	-	MVPA, physical activity score	Fitness: sign. inverse association between overweight and standing long jump (p=0.001) and overweight and CRF (p=0.001). sign. inverse association between overweight and 50-m sprint (p=0.02). Adolescents with lower median score in the standing broad jump and predicted CRF: higher odds ratio to be overweight (OR=3.1 (1.7 to 5.8) or obese (OR=9.1 (3.4 to 24.1).<br>PA: sign. inverse association between BMI and PA score (p=0.006). Low PA: sign. higher odds ratio to be overweight or obese (overweight OR=4.6 (2.5 to 8.5), obese OR=10.8 (3.9 to 30.1).
Aires et al., 2010 [33]	longi-tudinal; age: 11–16 years; N=345	BMI (not categorized)	shuttle run	back saver, sit and reach, curl ups, push ups	-	sport outside school in (non) organized sport (MVPA), sedentary activities (TV, PC time)	Fitness: sign. neg. association between BMI and fitness level (p<0.001). High fitness level at baseline: lowest pos. changes in BMI over all measurements; low fitness at baseline: increased BMI.<br>Low fitness level at baseline: neg. changes in total PAI; pos. changes in BMI.<br>PA: no analyses.

Sign. = significant; PA = physical activity; CRF = cardiorespiratory fitness; BMI = body mass index; MPA = moderate physical activity; MVPA = moderate-to-vigorous physical activity; VPA = vigorous physical activity; VVPA = very vigorous physical activity, WC = waist circumference; WHR = waist to hip ratio; LTPA = leisure-time physical activity; SSF = skinfold thickness; STF = truncal subcutaneous fat; PWC = physical work capacity

Reference	Study design	Anthropo-metrics	Cardio-respiratory fitness	Motor fitness	Objective physical activity	Subjective physical activity	Results
He et al., 2011 [5]	longi-tudinal; age: 8–13 years (T1); T2: 18 month later N= 2,179 (T1); N=1,795 (T2)	BMI [44]	shuttle run	-	-	categorized into physical active / inactive	Fitness: CRF: sign. inverse correlation with BMI in both surveys (r=-0.73 and r=-0.74; p<0.001): CRF inversely associated with the changes in BMI over the study period (ß=-0.63; p<0.001). Sign. higher CRF in normal weight or in physically active children (p<0.001).<br>PA: no sign. associations between PA and the changes in BMI.

Sign. = significant; PA = physical activity; CRF = cardiorespiratory fitness; BMI = body mass index; MPA = moderate physical activity; MVPA = moderate-to-vigorous physical activity; VPA = vigorous physical activity; VVPA = very vigorous physical activity, WC = waist circumference; WHR = waist to hip ratio; LTPA = leisure-time physical activity; SSF = skinfold thickness; STF = truncal subcutaneous fat; PWC = physical work capacity
