# Supplementary material for: The relationship between physical activity, physical fitness and overweight in adolescents: a systematic review of studies published in or after 2000
Source: BMC Pediatr. 2013 Feb 1;13:19. doi: 10.1186/1471-2431-13-19 (PMC3571910; doi:10.1186/1471-2431-13-19)
Supplement: Additional file 2 — Table S2. Overview of the strength of the different associations. Table 2 gives an overview of the different effects from physical activity and physical fitness on overweight and the interaction between physical activity, physical fitness and overweight respectively from each study. [file 1471-2431-13-19-S2.pdf]

Table 2: Overview of the strength of the different associations

Author	physical activity - overweight	gender differences	physical fitness - overweight	gender differences	Interaction PA, fitness, overweight	gender differences
Deforche et al., 2003 [31]	0	*	3	***		
Ng et al. [30]	0	n.a.	3	X		
Pate et al., 2006 [25]			3	X		
Ara et al., 2007 [29]	0	X	2	**		
Haerens et al., 2007 [8]	1	X	3	*		
Ortega et al., 2007 [26]	0	*	1	X	n.a. (data not shown)	n.a.
Fogelholm et al., 2008 [6]	0	n.a.	2	X	n.a. (overweight = mediator)	X
Lohman et al., 2008 [32]	1	n.a.	1	n.a.	1 (PA = moderator)	n.a.
Aires et al., 2010 [7]	0	n.a.	1	n.a.		
Huotari et al., 2010 [27]			n.a.	*	BMI & PA = predictors of CRF [a]	X
Ortega et al., 2010 [9]	0	n.a.	2	X	3 (CRF = moderator)	X
Gonzales-Suarez et al., 2011 [28]	0	X	2	n.a.		
Aires et al., 2010 [33]	1	n.a.	2	***	1 (CRF = mediator)	*
He et al., 2011 [5]	0	***	2	***		

3=strong effect; 2=moderate effect; 1=low effect; 0=no effect; ***=large gender differences; **=middle gender differences; *=small gender differences; X=no gender differences; n.a.=not available

[a] this study did not analyze the association between PA – overweight or fitness – overweight → no information about mediator or moderator effect, so that this study was not added to interaction studies in the manuscript
